# Genetic and Clinical Analyses of 13 Chinese Families With Cystine Urolithiasis and Identification of 15 Novel Pathogenic Variants in *SLC3A1* and *SLC7A9*

**DOI:** 10.3389/fgene.2020.00074

**Published:** 2020-02-18

**Authors:** Chuangye Li, Yongjia Yang, Yu Zheng, Fang Shen, Li Liu, Yanfang Li, Liping Li, Yaowang Zhao

**Affiliations:** ^1^Department of Urology, Hunan Children’s Hospital, Changsha, China; ^2^The Laboratory of Genetics and Metabolism, Hunan Children’s Research Institute (HCRI), Hunan Children's Hospital, University of South China, Changsha, China

**Keywords:** cystine urolithiasis, *SLC3A1*, *SLC7A9*, pathogenic variants, Chinese population

## Abstract

**Background:**

Cystinuria is a rare genetic disorder characterized by defective renal reabsorption of cystine, ornithine, arginine, and lysine. The increased urinary excretion of cystine results in the development of cystine urolithiasis (CU). The mutated *SLC3A1* and *SLC7A9* genes are the cause of CU, a global disorder. Its frequency and mutation spectrum vary between different populations. In Asia, the data for CU are limited.

**Method:**

Urinary stones were collected from patients of a single center over a five-year period and analyzed *via* Fourier transform infrared spectroscopy. Genomic DNA was isolated from 13 patients with CU and their parents and from 26 controls affected by calcium oxalate dihydrate stones. The coding regions and the exon–intron boundaries of *SLC3A1* and *SLC7A9* were subjected to PCR amplification and then sequenced *via* traditional Sanger sequencing. Genetic variants were functionally annotated using the InterVar, ClinVar, gnom AD, and HGMD databases.

**Results:**

From the 232 samples of urinary stones, we identified 13 patients with CU (10 males and 3 females). The onset age was from 7 months to 9 years. The CU stones varied from 0.26 cm^3^ to 18.67 cm^3^. Sanger sequencing detected a total of 14 *SLC3A1* (nine were novel) and 10 *SLC7A9* (six were novel) rare variants from the 13 CU families. All variants, including 15 novel variants, were pathogenic, disease-causing, or damaging.

**Conclusion:**

All 13 pediatric CU families harbored *SLC3A1* or/and *SLC7A9* rare variants. A total of 15 novel pathogenic variants in *SLC3A1* and *SLC7A9* were identified. This study expanded the known mutational spectrum of CU in the Chinese population.

## Background

Urolithiasis, also known as urinary calculi disease, develops when a solid piece of material exists in the urinary tract (Miller and Lingeman, 2007). Most kidney stones form because of a combination of genetics and environmental factors (Miller and Lingeman, 2007). Urolithiasis have serious consequences if not treated immediately. Cystine urolithiasis (CU) is a genetic disorder characterized by defective renal reabsorption of cystine, arginine, lysine, and ornithine; the increased urinary excretion of cystine results in the formation of kidney stones (Eggermann et al., 2012; Fattah et al., 2014). Two genes responsible for cystinuria and CU have been identified: *SLC3A1*, which encodes the heavy subunit rBAT of the renal b0,+ transporter; and *SLC7A9*, which encodes b0,+AT, the interacting light subunit of the b0,+ transporter (Calonge et al., 1994; Feliubadaló et al., 1999). b0,+ and b0,+AT combine with each other through disulfide linkage, and only the combined b0,+–b0,+AT heterodimer is functional (Chillarón et al., 2010). The inheritance for cystinuria may vary from autosomal recessive (AR) to autosomal dominant (AD) with incomplete penetrance to digenic pattern (Dello Strologo et al., 2002; Sahota et al., 2019). Accordingly, cystinuria is divided into the following subtypes: biallelic *SLC3A1* mutations (AA genotype), biallelic *SLC7A9* mutations (BB genotype), a single heterozygous *SLC3A1* mutation (A genotype), a single heterozygous *SLC7A9* mutation (B genotype), and a single heterozygous *SLC3A1* mutation combined with a single heterozygous *SLC7A9* mutation (AB genotype) (Dello Strologo et al., 2002; Sahota et al., 2019).

Cystinuria is a global disease with a population-specific prevalence (Eggermann et al., 2012). Although the overall frequency is estimated at 1:7,000 in neonates (Segal and Thier, 1995), the frequency varies in different areas. The rate of CU is 1:2,500 in Libyan Jews, 1:15,000 in Americans, and 1:100,000 in Swedes (Weinberger et al., 1974; Segal and Thier, 1995). In Libyan Jews, the mutant gene carrier rate is as high as 1:25 (Weinberger et al., 1974). In Sweden, 0.5% of the general population carries the *SLC3A1* p.Met467Thr variant (Harnevik et al., 2001; Harnevik et al., 2003). In Asia, cystinuria is rarely reported and its frequency in the Chinese population is unclear.

In this study, we analyzed the composition of 232 samples of kidney stones and subjected 13 cases of childhood CU to genetic analysis. A total of 24 pathogenic variants in *SLC3A1* and *SLC7A9* were detected, 15 of which were novel. These data expanded the spectrum of CU variants in the Chinese population.

## Methods

### Criteria for Pediatric CU and the Controls

Data from pediatric CU patients were collected from 2012 to 2017. A total of 13 CU patients were enrolled in this study (**Supplementary Data 1**). Patients were diagnosed with pediatric CU if (i) they were aged 0–18 years old, (ii) passed visible urinary stones under a clinical setting, and (iii) passed pure or cystine stones as identified *via* stone composition analysis. The 26 controls were taken from sporadic cases of patients with calcium oxalate dihydrate stones (**Supplementary Data 1**).

### DNA Isolation and Sanger Sequencing

Peripheral blood samples were obtained from 13 Trios-families and 26 sporadic cases of other kidney stones (calcium oxalate dihydrate stones). DNA was extracted using a genomic DNA isolation kit (D3392-02; Omega Bio-Tek, Inc., Norcross, GA, USA) in accordance with a standard protocol. The coding regions and intron–exon junctions of the *SLC3A1* and *SLC7A9* genes were amplified *via* PCR by using primers synthesized by BGI, a local biotech company (Shenzhen, China). Primers were designed with Primer3 software (http://frodo.wi.mit.edu). The primer sequences and PCR conditions are provided in **Supplementary Table 1**. Sanger sequencing was performed on an Applied Biosystems™ 3500 Dx Genetic Analyzer by using a BigDye 3.1 sequencing kit (Applied Biosystems, CA, USA).

### Variant Validation

All Sanger sequencing results were compared with the reference *SLC3A1* and *SLC7A9* sequences (NM_000341 and NM_014270, GRCh37/hg19, http://genome.ucsc.edu/cgi-bin/hgGateway) by applying SEQMAN software (DNA Star Package, Wisconsin, USA). Genetic variants were functionally annotated using InterVar (version 20180118) and screened for reported mutations by using ClinVar (version 20180603) and the HGMD database (public version). We further customized the clinical classification of each variant in accordance with the ACMG/AMP 2015 guidelines by applying automatically interpreted InterVar results and by integrating individualized information, such as family history and inheritance mode, obtained in this study with the results of previous studies.

## Results

### Clinical Data of 13 Patients With Pediatric CU

A total of 13 patients with CU were identified, 10 of whom were males and 3 were females. These patients originated from 232 patients with urinary stones that we treated from 2002 to 2007. The patients had a mean age of 3.82 ± 3.12 years (range: 0.6–9.3) at first stone onset. The mean stone size of the patient group was 4.12 ± 4.96 cm^3^ (range: 0.26–18.67). Two urethral (3.77%), 2 bladder (3.77%), 14 ureteral (26.42%), 22 renal pelvic or upper/middle calyceal (41.51%), and 13 lower calyceal (46.43%) calculi were noted. Five children had bilateral calculi, whereas two children had staghorn calculi. Seven children had a family history of CU. Twelve patients had normal initial renal function (serum creatinine [CREA] 20–120 μmol/L, blood urea nitrogen [BUN] 1.8–8.2 mmol/L, and serum uric acid [UA] 90–350 μmol/L). The remaining patient with bilateral ureteral calculi exhibited various degrees of renal dysfunction (625.3, 36.82, and 618.8 μmol/L). The mean number of operations was 3.61 ± 2.14 (range: 1–9), including 11 minimally invasive percutaneous nephrolithotomy, 10 retrograde intrarenal surgery, and 26 ureteroscopic lithotripsy. The mean stone-free rate was 46.15% after the final operation. **Table 1** lists the clinical features of the 13 patients with CU.

**Table 1:** Clinical features of 13 patients with cystine urolithiasis.

Characteristics	Patient 1	Patient 2	Patient 3	Patient 4	Patient 5	Patient 6	Patient 7	Patient 8	Patient 9	Patient 10	Patient 11	Patient 12	Patient 13
Gender	Male	Male	Male	Male	Female	Male	Male	Female	Male	Male	Male	Male	Female
Age, yr	9.3	1.7	1.8	1	3.1	2.8	1.5	8.3	9.3	2.9	0.6	2.8	4.6
Initial presentation	Stone	Stone	UTI	GHU	Stone	Stone	Stone	GHU	Stone	Stone	Stone	Stone	GHU
Bilateral stones	-	+	+	-	-	-	-	-	-	+	+	+	-
Staghorn stone	+	-	-	-	-	-	-	-	-	+	-	-	-
Number of stones	6	2	3	4	2	2	1	3	2	14	9	2	3
Stone size(cm^3^)	7.75	2.83	2.50	1.18	1.28	3.41	7.77	0.26	3.10	18.67	1.17	2.51	1.17
Number of operations	3	6	1	2	3	3	1	3	9	5	3	4	4
MPCNL	2	3	0	0	0	0	0	2	0	4	0	0	0
RIRS	1	0	0	1	1	1	0	1	3	0	0	0	2
URL	0	3	1	1	2	2	1	0	6	1	3	4	2
Family history	-	+(Uncle)	-	+(Parents)	-	+ (Brother)	+ (Brother)	+(Great grand-father)	+(Father)	-	-	+(Great-uncle)	-
Urolithiasis at last follow-up	-	-	+	+	-	+	-	+	+	+	-	-	+
Infant feeding	Sanlu milk powder	Breast milk supplemented with milk	Breast milk	Breast milk for 2 months,goat's milk thereafter	Breast milk supplemented with milk	Milk powder	Breast milk	Breast milk	Milk	Breast milk for 3 months, milk thereafter	Milk	Breast milk	Breast milk for 3 months, milk thereafter
Comorbidity	-	-	-	-	-	-	-	-	-	-	-	-	-
Serum calcium	2.3	2.36	2.29	2.51	2.35	2.38	2.21	2.35	2.41	2.24	2.24	2.19	2.33

Sanlu milk powder was found to contain high levels of melamine. GHU, gross hematuria; MPCNL, minimally invasive percutaneous nephrolithotomy; RIRS, retrograde intrarenal surgery; URL, ureteroscopic lithotripsy.

### Identification of *SLC3A1* and *SLC7A9* Variants on 24 Alleles *via* Sanger Sequencing

**Figure 1:**
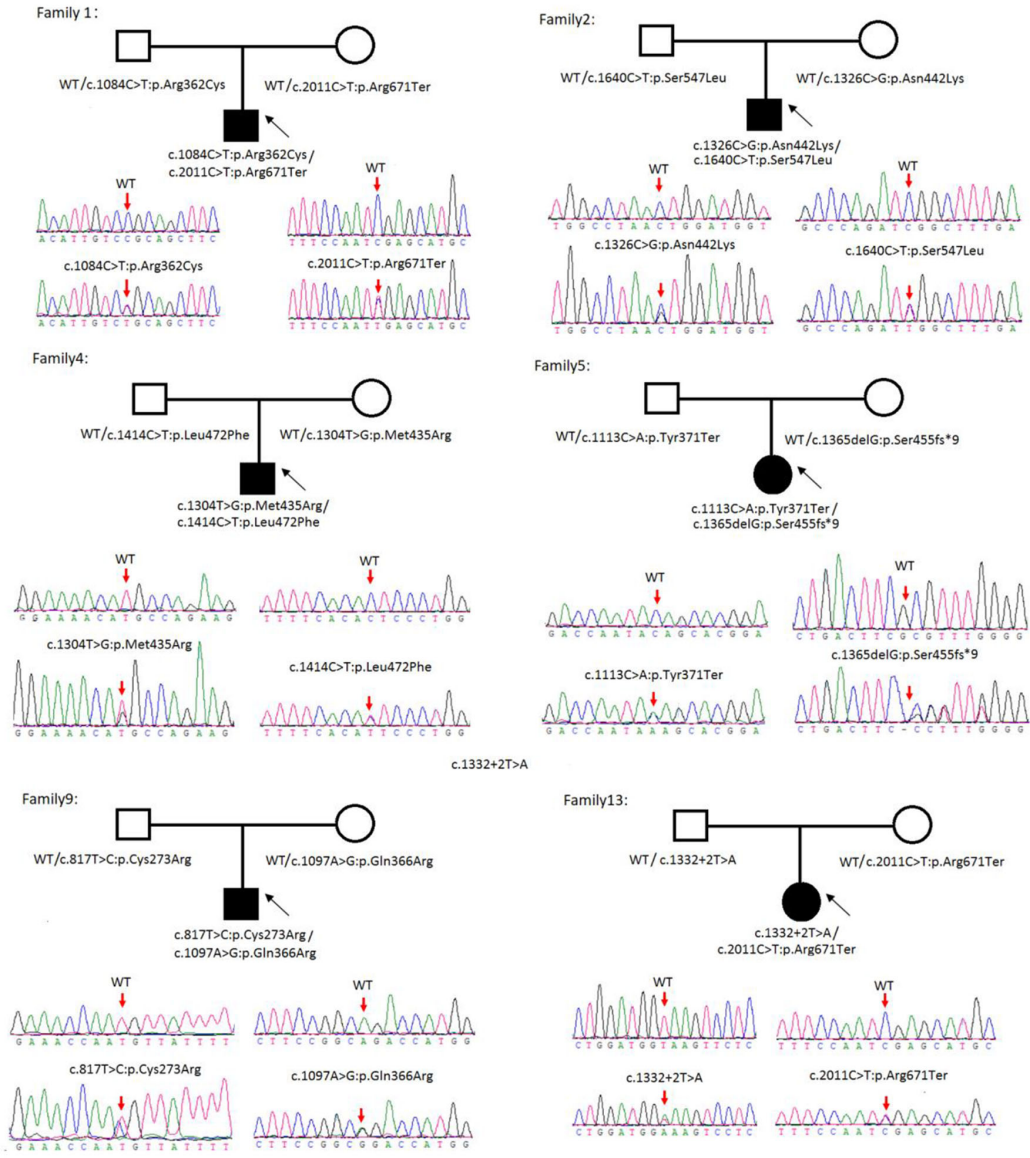
Rare variants of AA genotypes on six cystine urolithiasis (CU) families. Solid squares or circles mean the patients were affected by CU. Open squares or circles denote that the patients were not affected by CU.

**Figure 2:**
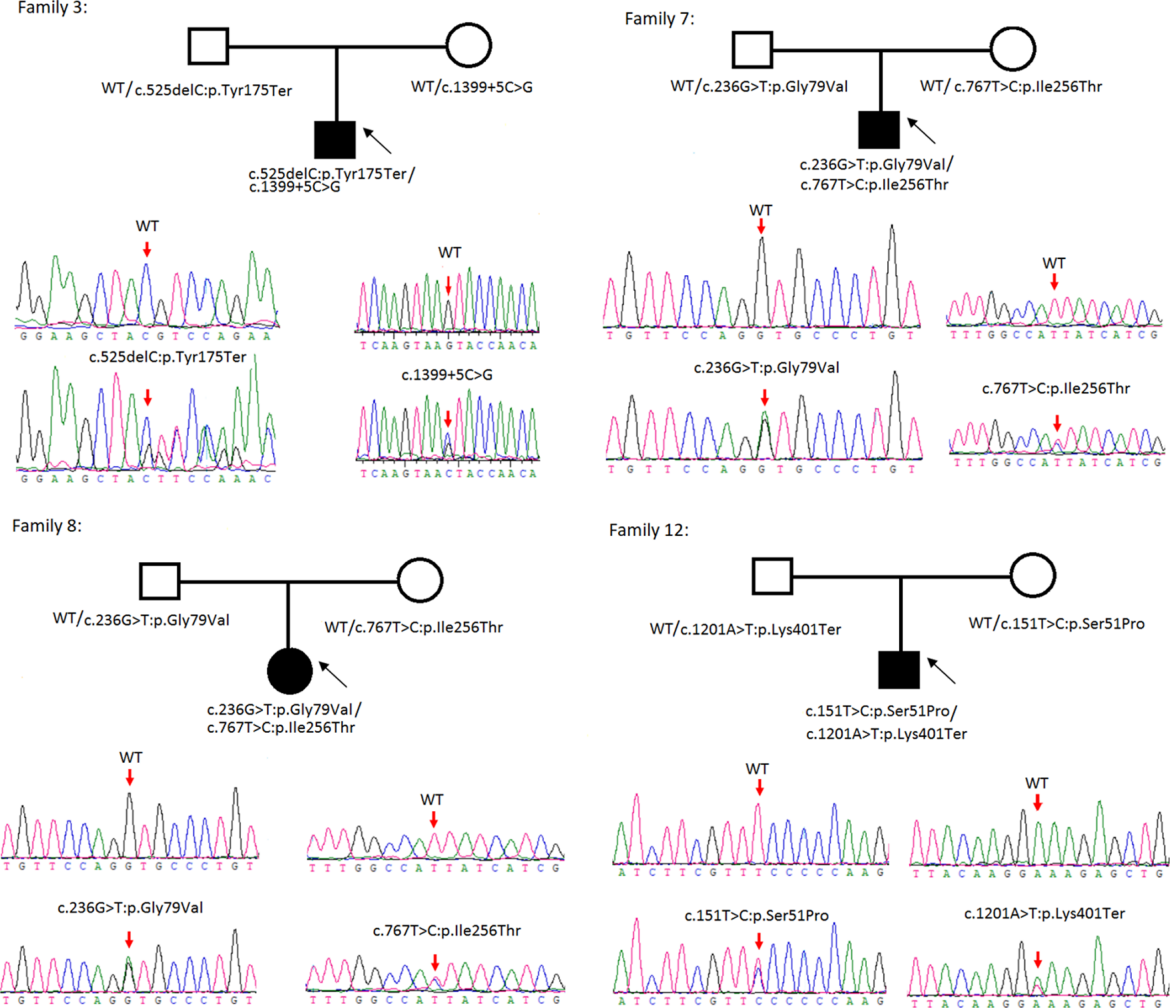
Rare variants of BB genotypes on four cystine urolithiasis (CU) families. Solid squares or circles mean the patients were affected by CU. Open squares or circles denote that the patients were not affected by CU.

**Figure 3:**
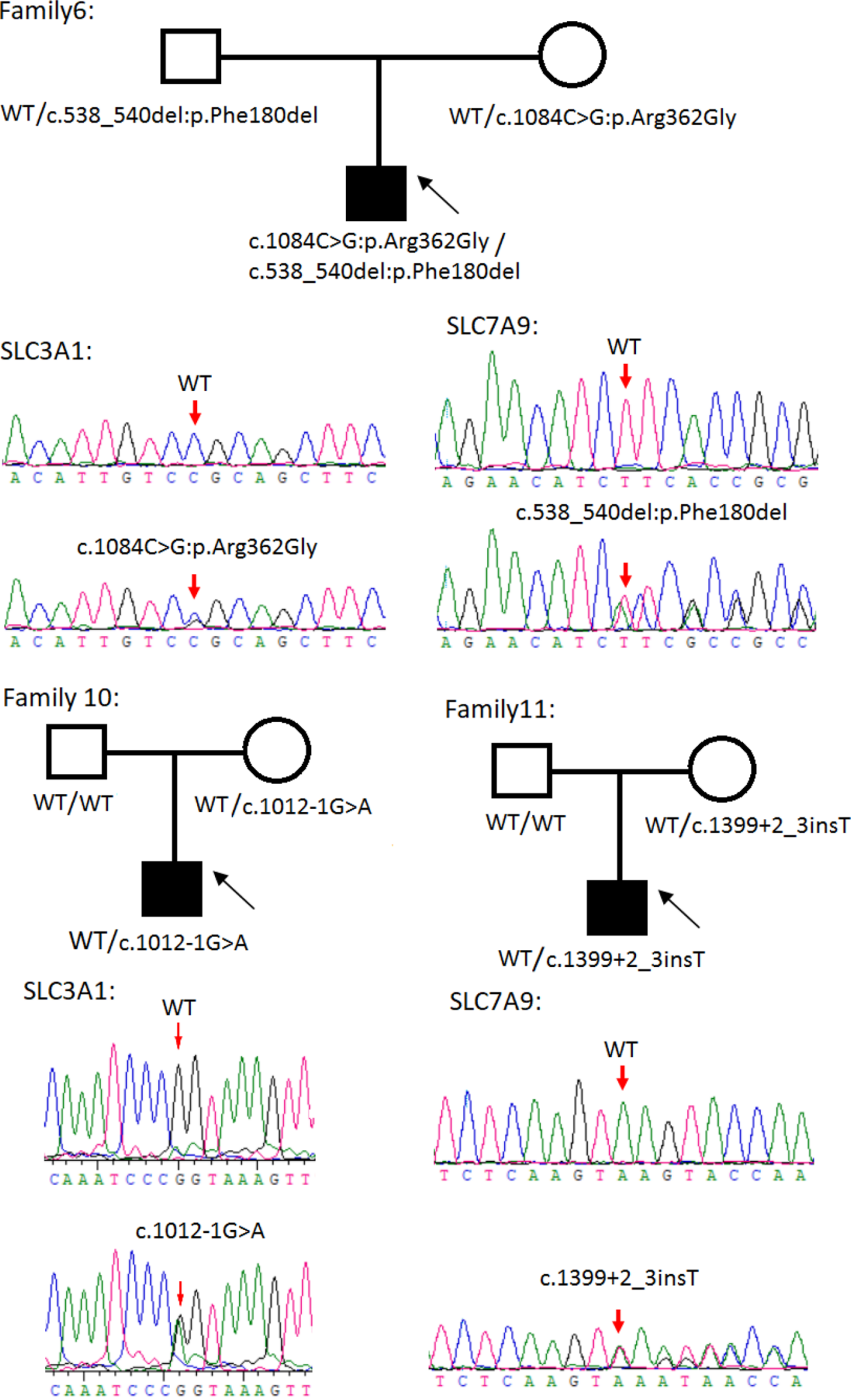
Rare variants of AB, A or B genotypes in three cystine urolithiasis (CU) families. Solid squares or circles mean the patients were affected by CU. Open squares or circles denote that the patients were not affected by CU.

Sanger sequencing was successfully conducted for the 13 patients with pediatric CU and their parents. After filtering out variants with MAF > 0.01 using the gnomAD database and the variants present in 26 controls, we detected 14 *SLC3A1* and 8 *SLC7A9* variants on 24 alleles from these 13 families (**Table 2**, **Figures 1**–**3**). These variants covered the whole spectrum of mutations and ranged from insertion, deletion, splicing, and missense variants. Six families presented with biallelic *SLC3A1* variants (AA genotype) (**Figure 1**), and four families exhibited biallelic *SLC7A9* variants (BB genotype) (**Figure 2**). The genotypes of the remaining three families included a single heterozygous *SLC3A1* variant (A genotype), a single heterozygous *SLC7A9* variant (B genotype), and a single heterozygous *SLC3A1* variant combined with a single heterozygous *SLC7A9* variant (AB genotype) (**Figure 3**).

**Table 2:** Rare variants on *SLC3A1* AND *SLC7A9* detected in 13 families with cystine urolithiasis.

Patient	Position	Ref	Alt	Gene	Func.refGene		Exonic Func.	AA Change	HGMD	ACMG interpretation
1	44528214	C	T	SLC3A1	exonic	NM_000341	nonsynonymous SNV	exon6:c.1084C > T:p.Arg362Cys	Pathogenic, ID : CM961306	Pathogenic (PS1,PM1,PM2,PM3,PP3,PP4)
1	44547731	C	T	SLC3A1	exonic	NM_000341	stopgain	exon10:c.2011C > T:p.Arg671Ter	NA	Pathogenic (PVS1,PM2,PM3,PP3,PP4)
2	44531471	C	G	SLC3A1	exonic	NM_000341	nonsynonymous SNV	exon7:c.1326C > G:p.Asn442Lys	NA	Likely pathogenic (PM2,PM3,PP4)
2	44547360	C	T	SLC3A1	exonic	NM_000341	nonsynonymous SNV	exon10:c.1640C > T:p.Ser547Leu	Pathogenic, ID : CM014096,CM034062,CM070067	Pathogenic (PS1,PM1,PM2,PM3,PP3,PP4)
3	33353446	G	–	SLC7A9	exonic	NM_001126335	stopgain	exon5:c.525delC:p.Tyr175Ter	NA	Pathogenic (PVS1,PM2,PP3,PP4)
3	33324050	C	G	SLC7A9	intronic	NM_001126335	splicing	Intron12:c.1399+5C > G	NA	Likely pathogenic (PM2,PM3,PP3,PP4)
4	44531449	T	G	SLC3A1	exonic	NM_000341	nonsynonymous SNV	exon7:c.1304T > G:p.Met435Arg	NA	Likely pathogenic (PM1,PM2,PM3,PP3,PP4)
4	44539806	C	T	SLC3A1	exonic	NM_000341	nonsynonymous SNV	exon8:c.1414C > T:p.Leu472Phe	Pathogenic, ID: HM070022	Pathogenic (PS1,PM1,PM2,PM3,PP3,PP4)
5	44528243	C	A	SLC3A1	exonic	NM_000341	stopgain	exon6:c.1113C > A:p.Tyr371Ter	NA	Pathogenic (PVS1,PM2,PM3,PP4)
5	44539757	G	–	SLC3A1	exonic	NM_000341	frameshift deletion	exon8:c.1365delG:p.Ser455fs	NA	Pathogenic (PVS1,PM2,PM3,PP4)
6	44528214	C	G	SLC3A1	exonic	NM_000341	nonsynonymous SNV	exon6:c.1084C > G:p.Arg362Gly	Pathogenic, ID: CM961306	Pathogenic (PS1,PM1,PM2,PP3,PP4)
6	33353431	GAA	–	SLC7A9	exonic	NM_014270	nonframeshift deletion	c.538_540del:p.Phe180del	NA	Likely pathogenic (PM2,PM3,PM4,PP4)
7	33355244	C	A	SLC7A9	exonic	NM_014270	nonsynonymous SNV	exon4:c.236G > T:p.Gly79Val	NA	Uncertain significance (PM2,PP3,PP4)
7	33350853	A	G	SLC7A9	exonic	NM_014270	nonsynonymous SNV	exon8:c.767T > C:p.Ile256Thr	NA	Uncertain significance (PM2,PP3,PP4)
8	33355244	C	A	SLC7A9	exonic	NM_014270	nonsynonymous SNV	exon4:c.236G > T:p.Gly79Val	NA	Uncertain significance (PM2,PP3,PP4)
8	33350853	A	G	SLC7A9	exonic	NM_014270	nonsynonymous SNV	exon8:c.767T > C:p.Ile256Thr	NA	Uncertain significance (PM2,PP3,PP4)
9	44513222	T	C	SLC3A1	exonic	NM_000341	nonsynonymous SNV	exon4:c.817T > C:p.Cys273Arg	NA	Likely Pathogenic (PM1,PM2,PM3,PP3,PP4)
9	44528227	A	G	SLC3A1	exonic	NM_000341	nonsynonymous SNV	exon6:c.1097A > G:p.Gln366Arg	NA	Likely Pathogenic (PM1,PM2,PM3,PP3,PP4)
10	44527229	G	A	SLC3A1	exonic	NM_000341	splicing	exon5:c.1012-1G > A	Pathogenic, ID: CS983898	Pathogenic (PS1,PM2,PP3,PP4)
11	33324053	–	A	SLC7A9	splicing	NM_014270	splicing	intron12:c.1399+2_3insT	Pathogenic, ID: CI060712)	Pathogenic (PVS1,PM2,PP4) | Uncertain significance (PM2,PP4)
12	33355619	A	G	SLC7A9	exonic	NM_014270	nonsynonymous SNV	exon3:c.151T > C:p.Ser51Pro	Pathogenic, ID: CD050153)	Pathogenic (PS1,PM2,PM3,PP3,PP4)
12	33333097	T	A	SLC7A9	exonic	NM_014270	stopgain	exon11:c.1201A > T:p.Lys401Ter	Pathogenic,p.Lys401Glu, ID: HM070035)	Pathogenic (PVS1,PS1,PM2,PM3,PP3,PP4)
13	44531479	T	A	SLC3A1	splicing	NM_000341	splicing	Intron7:c.1332+2T > A	NA	Pathogenic (PVS1,PM2,PM3,PP3,PP4)
13	44547731	C	T	SLC3A1	exonic	NM_000341	stopgain	exon10:c.2011C > T:p.Arg671Ter	NA	Pathogenic (PVS1,PM2,PM3,PP3,PP4)

Among these 24 variants, 15 have not been reported elsewhere (9 on *SLC3A1*, 6 on *SLC7A9*).

According to the ACMG/AMP 2015 guideline classification (**Table 2**), five of the nine *SLC3A1* variants were pathogenic and four were likely pathogenic; two of the six *SLC7A9* variants were pathogenic and another two were likely pathogenic. The classifications of the remaining two variants of *SLC7A9* were uncertain. These 15 novel variants included the following: (1) For *SLC3A1*: c.2011C > T:p.Arg671Ter, c.1326C > G:p.Asn442Lys, c.1304T > G:p.Met435Arg, c.1113C > A:p.Tyr371Ter, c.1365delG:p.Ser455Valfs, c.817T > C:p.Cys273Arg, c.1097A > G:p.Gln366Arg, c.1332+2T > A, and c.2011C > T:p.Arg671Ter. (2)For *SLC7A9*: c.525delC:p.Tyr175Ter, c.1399+5C > G,c.538_540del:p.180_180del, c.236G > T:p.Gly79Val, c.767T > C:p.Ile256Thr, and exon11:c.1201A > T:p.Lys401Ter.

Further functional effects were tested for all variants *via* three programs in silico (i.e., REVEL, ClinPred, and MutationTaster). Results showed that 13 of the 15 novel variants were damaging, disease-causing, or pathogenic (**Table 3**).

**Table 3:** Effect predictions of rare variants by three in silico software. REVEL: V0.21.0 (http://revel.github.io/); ClinPred (Alirezaie et al., 2018); MutationTaster (Schwarz et al., 2014).

Gene	Variant	Effect	REVEL	ClinPred	MutationTaster
SLC3A1	exon6:c.1084C > T:p.Arg362Cys	nonsynonymous	Damaging	pathogenic	Disease_causing
SLC3A1	exon10:c.2011C > T:p.Arg671Ter	stopgain	–		Disease_causing
SLC3A1	exon7:c.1326C > G:p.Asn442Lys	nonsynonymous	Damaging	pathogenic	Disease_causing
SLC3A1	exon10:c.1640C > T:p.Ser547Leu	nonsynonymous	Damaging	pathogenic	Disease_causing
SLC7A9	exon5:c.525delC:p.Tyr175Ter	stopgain	–		Disease_causing
SLC7A9	Intron12:c.1399+5C > G	–	–		–
SLC3A1	exon7:c.1304T > G:p.Met435Arg	nonsynonymous	Damaging	pathogenic	Disease_causing
SLC3A1	exon8:c.1414C > T:p.Leu472Phe	nonsynonymous	Damaging	pathogenic	Disease_causing
SLC3A1	exon6:c.1113C > A:p.Tyr371Ter	stopgain	–		Disease_causing
SLC3A1	exon8:c.1365delG:p.Ser455fs	frameshift	–		Disease_causing
SLC3A1	exon6:c.1084C > G:p.Arg362Gly	nonsynonymous	Damaging	pathogenic	Disease_causing
SLC7A9	c.538_540del:p.Phe180del	–	–		–
SLC7A9	exon4:c.236G > T:p.Gly79Val	nonsynonymous	Damaging	pathogenic	Disease_causing
SLC7A9	exon8:c.767T > C:p.Ile256Thr	nonsynonymous	Damaging	pathogenic	Disease_causing
SLC7A9	exon4:c.236G > T:p.Gly79Val	nonsynonymous	Damaging	pathogenic	Disease_causing
SLC7A9	exon8:c.767T > C:p.Ile256Thr	nonsynonymous	Damaging	pathogenic	Disease_causing
SLC3A1	exon4:c.817T > C:p.Cys273Arg	nonsynonymous	Damaging	pathogenic	Disease_causing
SLC3A1	exon6:c.1097A > G:p.Gln366Arg	nonsynonymous	Damaging	pathogenic	Disease_causing
SLC3A1	exon5:c.1012-1G > A	splicing	–	–	Disease_causing
SLC7A9	intron12:c.1399+2_3insT	splicing	–		Disease_causing
SLC7A9	exon3:c.151T > C:p.Ser51Pro	nonsynonymous	Damaging	pathogenic	Disease_causing
SLC7A9	exon11:c.1201A > T:p.Lys401Ter	stopgain	–	–	Disease_causing
SLC3A1	Intron7:c.1332+2T > A	splicing	–		Disease_causing
SLC3A1	exon10:c.2011C > T:p.Arg671Ter	stopgain	–		Disease_causing

The remaining two *SLC7A9* variants (i.e., c.1399+5C > G and c.538_540del:p.Phe180del) were likely pathogenic. Thus, further confirmatory experiments are needed.

### Genotype-Phenotype Correlation for 13 CU Families

The 13 CU families were divided into three groups: classical AR inheritance (six AA and four BB genotypes), AD inheritance (one A and one B genotype), and digenic inheritance (one AB genotype) (**Table 4**). Several points were observed in this study: (1) *SLC3A1* is the main disease gene for childhood CU. *SLC3A1* is mutated in 8 out of the 13 patients with CU and the number of stones is 2–9 for AA genotype and 1–3 for BB genotype. (2) Patients with AD inheritance had the highest number of stones. In family number 10 with A genotype, the number of stones is 14. In family number 11 with B genotype, the number of stones is 9. (3) Childhood CU may represent a more aggressive form. By contrast, the pathogenic variants in *SLC3A1* or *SLC7A9* (Eggermann et al., 2012) cannot all be detected in cystiruia patients. In this study, we detected the pathogenic variants in *SLC3A1* or *SLC7A9* in all CU patients, suggesting that pediatric CU patients may represent a more aggressive group.

**Table 4:** In-brief lists of genotype and phenotype data for 13 families with cystine urolithiasis.

Inheritance	Genotype	Number of patients	Onset age	Stone size	Stone number
AR	AA	6	1-9.3	1.18-7.75	2-9
	BB	4	1.5-8.3	0.26-7.77	1-3
AD	A	1	2.9	18.67	14
	B	1	0.6	1.17	9
Digenic	AB	1	2.8	3.41	2

AD, autosomal dominant; AR, autosomal recessive; Onset age: Years; Stone size: cm^3^.

### Phenotypic Details for Three CU Patients With Heterozygous Variants

Three cases harboring *SLC3A1* or (and) *SLC7A9* heterozygous variants were identified. For patient number 6 with AB genotype, no clinical symptom of urolithiasis was observed during health examinations. The patient's 24-h urinary calcium was 0.42 mmol/L, 24-h urinary phosphorus was 9.75 mmol/L, serum calcium was 2.38 mmol/L, serum phosphate was 1.61 mmol/L, BUN was 3.39 mmol/L, CREA was 18.32 µmol/L, and UA was 269.31 µmol/L. However, the B ultrasound detected stones in the patient's urinary system.

Patient number 10 with A genotype carried an *SLC3A1* spicing variant (c.1012-1G > A) that was previously reported as pathogenic (Saadi et al., 1998). The patient's initial symptom was dysuria but without urinary frequency and urgency. His 24-h urinary calcium was 0.12 mmol/L, 24-h urinary phosphorus was 7.94 mmol/L, serum calcium was 2.24 mmol/L, BUN was 4.42 mmol/L, CREA was 41.65 µmol/L, and serum UA was 182.91 µmol/L.

Patient number 11 with B genotype carried an *SLC7A9* spicing variant (1399 + 2_3insT) that was also previously reported as pathogenic (Yuen et al., 2006). The patient had spontaneous discharge of small stones (at 3 months old) according to his parents. The patient's 24-h urinary calcium was 0.59 mmol/L, 24-h urinary phosphorus was 3.92 mmol/L, serum calcium was 2.24 mmol/L, serum phosphate was 2.19 mmol/L, BUN was 3.37 mmol/L, CREA was 32.2 µmol/L, and serum-UA was 217.26 µmol/L.

## Discussion

Cystinuria is a metabolic disease caused by defects in the *SLC3A1* and *SLC7A9* genes. These gene defects result in decreased renal reabsorption of cystine, ornithine, arginine, and lysine. Most patients with cystinuria develop CU, but the age at onset varies (Harnevik et al., 2003). In this study, we subjected 13 patients with pediatric CU to mutation screening and observed typical *SLC3A1* and *SLC7A9* pathogenic variants consistent with AA, BB, or AB genotypes in 11 of the families with CU and heterozygous *SLC3A1* and *SLC7A9* rare variants in two of the families with CU (i.e., family numbers 10 and 11). Family numbers 10 and 11 suffer from c.1012-1G > A and 1399 + 2_3insT splicing variants, respectively. These two variants were reported previously as pathogenic (Harnevik et al., 2001; Harnevik et al., 2003). However, no CU phenotypes were observed for both maternal carriers. This situation may be indicative of the non-full penetrance for the heterozygous mutation in females.

According to the ACMG/AMP 2015 guidelines or the predictions by the software in silico, all 24 rare variants are pathogenic, likely pathogenic, disease-causing, or damaging (**Tables 2** and **3**). This result is similar to that obtained by another study (Shen et al., 2017). Shen et al. (2017) examined 13 pediatric patients with CU and found that all patients harbored pathogenic *SLC3A1* or *SLC7A9* variants. These results raise the question of whether newborns with mutations in the *SLAC3A1* or *SLC7A9* genes should be afforded certain precautions, especially as next-generation sequencing has become widely available.

**Figure 4:**
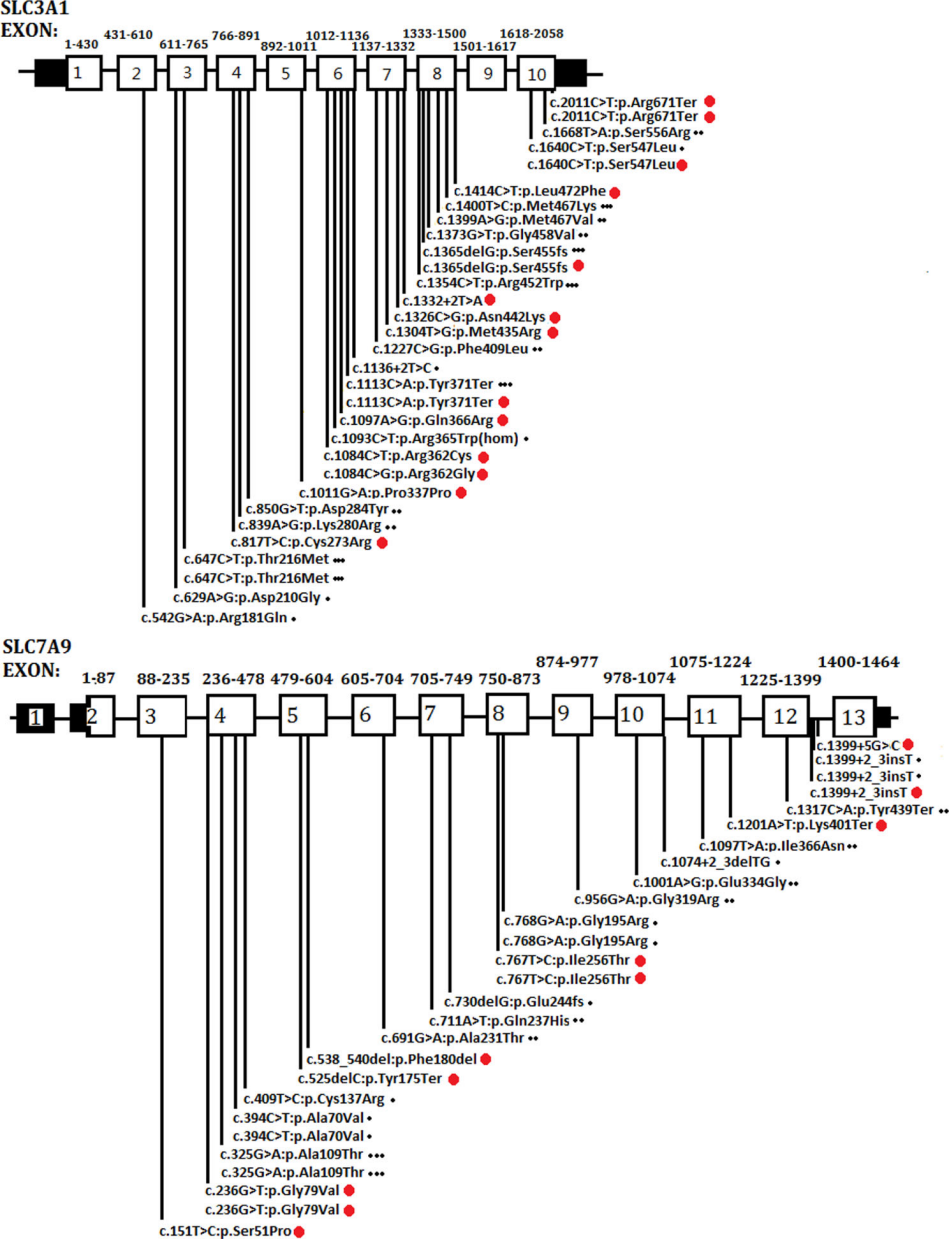
Schematic presentation of *SLC3A1* or *SLC7A9* pathogenic variants in the Chinese population that have been reported thus far. One dot (at the end of the variants) represents the variants reported by Yuen et al. (2006). Two dots denote the variants reported by Shen et al. (2017). Three dots mean the variants reported by Ma et al. (2018). Red dots are the variants identified in the present study.

As a global disorder, the prevalence of cystinuria varies in different populations, and hotspot mutations have been described previously. The typical mutation hotspot site is *SLC3A1* p.Met467Thr (Weinberger et al., 1974; Harnevik et al., 2001; Botzenhart et al., 2002; Eggermann et al., 2012). A study reported that 0.5% of the Swedish population carries the hotspot variant (Harnevik et al., 2001). China is the most populated country in the world. However, to the best of our knowledge, only four studies (including the present study) have been conducted in China concerning this topic. These studies involved 40 patients in total (Yuen et al., 2006; Shen et al., 2017; Ma et al., 2018). A total of 26 *SLC3A1* and 21 *SLC7A9* pathogenic variants have been reported in the Chinese population. As shown in **Figure 4**, the Chinese variants were evenly distributed through the *SLC3A1* or *SLC7A9* genes, and only five *SLC3A1* and six *SLC7A9* variants were reported twice. No *SLC3A1*-p.Met467Thr was detected in the Chinese patients (**Figure 4**). Therefore, the *SLC3A1* and *SLC7A9* variants in the patients likely represent mutation spectra that differ from those in other populations.

## Conclusions

We detected a total of 24 rare variants in the *SCL3A1* and *SLC7A9* genes from 13 families with CU. Among these variants, 15 were novel. Functional evaluation analysis confirmed that all these novel variants are pathogenic, damaging, or disease-causing. These data expanded the spectrum of CU variants in the Chinese population.

## Data Availability Statement

The SLC3A1 and SLC7A9 genetic polymorphism data can be found in ClinVar, accession number SUB6699202.

## Ethics Statement

The studies involving human participants were reviewed and approved by Academic Committee of Hunan Children's Hospital (Approval number: HCHLL58, Changsha City, Hunan Province, China). Written informed consent to participate in this study was provided by the participants' legal guardian/next of kin.

## Author Contributions

Writing of the first draft: CL, YY, YZ, and YwZ. Review and Critique: YY and YwZ. Conception: CL, YY, YwZ. Organization: YY, YZ. Execution: YY, CL, YwZ, YZ. Clinical support: FS, LL, YL, LpL. Material support: YY, YwZ, CL, YZ, YL and LL.

## Funding

This work was supported by the Natural Science Foundation of Hunan Province, China (2017JJ2139) and Hunan Health Commission Research Fund (B2019019).

## Conflict of Interest

The authors declare that the research was conducted in the absence of any commercial or financial relationships that could be construed as a potential conflict of interest.
