# Supplementary material for: Genetic and Clinical Analyses of 13 Chinese Families With Cystine Urolithiasis and Identification of 15 Novel Pathogenic Variants in SLC3A1 and SLC7A9
Source: Front Genet. 2020 Feb 18;11:74. doi: 10.3389/fgene.2020.00074 (PMC7040229; doi:10.3389/fgene.2020.00074)
Supplement: Supplementary file 1 [file DataSheet_1.docx]

**Supplementary data 1:**

**Results for analysis of pediatric kidney stone composition**

A total of 232 samples of urinary stones were collected from 181 male and 51 female patients in Hunan Children’s Hospital (Changsha City, China) over the period of June 2012 to June 2017. The age of the patients ranged from 0.6 years to 16 years. Among 232 cases, 101 (43.5%); 81 (34.9%), 35 (15.1%), and 15 (6.5%) had kidney, ureteral, bladder, and urethral stones, respectively. Pure stones were observed in 134 cases (57.8%). Calcium oxalate monohydrate stones were found in 63 cases (27.2%), ammonium urate stones in 27 cases (11.6%), calcium oxalate dihydrate stones in 16 cases (6.9%), cystine stones in 13 cases (5.6%), anhydrous uric acid stones in eight cases (3.4%), xanthine stones in three cases (1.3%), sodium urate monohydrate stones in two cases (0.9%), ammonium magnesium phosphate hexahydrate stones in one case (0.4%), and amorphic calcium phosphate stones in one case (0.4%). Mixed stones were observed in 98 cases (42.2%). Stones with calcium oxalate monohydrate, ammonium urate, calcium oxalate dihydrate, ammonium magnesium phosphate hexahydrate, carbonate apatite, anhydrous uric acid, or sodium urate monohydrate as the main component accounted for 43 cases (18.5%), 27 cases (11.6%), 16 cases (6.9%), six cases (2.6%), three cases (1.3%), two cases (0.9%), and one case (0.4%), respectively. The overall pediatric urinary stone composition is given in the following Figure.

**The method for analysis of kidney stone composition**

All stones were analyzed through Fourier transform infrared spectroscopy (FTIR, Medical device registration license in China: Shangdong-No.20142400080; Registered product standards: YZB/0685-2014). Stones were recovered through minimally invasive percutaneous nephrolithotomy, ureteroscopy, or spontaneous passage. The stones were washed with distilled water to remove attached blood and tissue and then completely dried in a hot air oven at 70 °C–100 °C. Each dry stone was crushed into homogeneous powder. Next, 1 mg of the stone powder was mixed and ground with 200 mg of potassium bromide powder by using an agate pestle and mortar until well blended. The mixture was pressed into a small round tablet, which was placed into the scanning chamber in the path of the FTIR spectrophotometer.


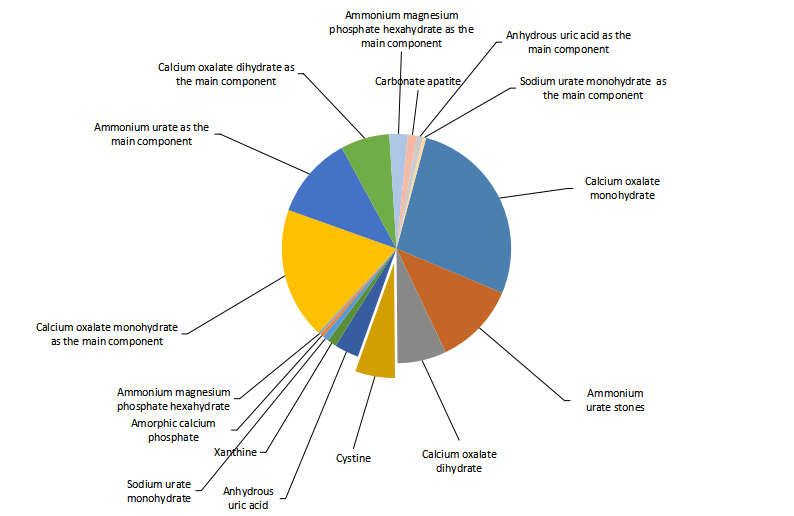


**Figure :** The overall urinary stone composition of 232 samples came from pediatric patients. Note: Pure stones and mixed stones are calculated together. Mixed stones are expressed by the main component.
