# Supplementary material for: Genetic and Clinical Analyses of 13 Chinese Families With Cystine Urolithiasis and Identification of 15 Novel Pathogenic Variants in SLC3A1 and SLC7A9
Source: Front Genet. 2020 Feb 18;11:74. doi: 10.3389/fgene.2020.00074 (PMC7040229; doi:10.3389/fgene.2020.00074)
Supplement: Supplementary file 2 [file Table_1.docx]

Supplementary Table 1: Primers and PCR condition for *SLC3A1* and *SLC7A9* genes amplification and mutation screening.

| **SLC3A1** | **Forward** | **Reverse** | **Product Size** | **Annealing temp.** |
| --- | --- | --- | --- | --- |
| EXON1 | 5'-ttccacctcccttactgcag-3' | 5'-aatctcatcctggcacccac-3' | 526bp | 62℃ |
| EXON2 | 5'-gaaccactacacccgaccta-3' | 5'-gagggagggagggaaagaaa-3' | 498bp | 60℃ |
| EXON3 | 5'-tgtattggggttacaggcgt-3' | 5'-cccctttgtaagtcagacagg-3' | 288bp | 60℃ |
| EXON4 | 5'-acctgctgctctctgtagaa-3' | 5'-atggagaagtctgtgtgggt-3' | 293bp | 60℃ |
| EXON5 | 5'-aggtgtgggagtcgctaaat-3' | 5'-aggctgagaaagaaaacactctg-3' | 399bp | 60℃ |
| EXON6 | 5'-cgtctcgcttggcttgag-3' | 5'-ggaaaggaatatggcaggcc-3' | 400bp | 55℃ |
| EXON7 | 5'-gcaagataagcagctgtgga-3' | 5'-ggcaacagcagcacattatg-3' | 318bp | 60℃ |
| EXON8 | 5'-tgctacgttgtgaactttctgt-3' | 5'-accatgattttcagcaatgca-3' | 372bp | 60℃ |
| EXON9 | 5'-agcagcagagatttcaagca-3' | 5'-ctgcaaggaagtctgtgattga-3' | 510bp | 60℃ |
| EXON10-1 | 5'-aggggcaaaattggagcaag-3' | 5'-cctttgtcggcagaattggt-3' | 387bp | 60℃ |
| EXON10-2 | 5'-acacaagagagctggatggc-3' | 5'-agcagttacattcaagctacaga-3' | 448bp | 60℃ |
| **SLC7A9** | **Forward** | **Reverse** | **Product Size** | **Annealing temp.** |
| EXON1 | 5'-ttgacctctgtaccttctgga-3' | 5'-tcaagcagtccttccacctc-3' | 373bp | 60℃ |
| EXON2 | 5'-actgactttgactctggggc-3' | 5'-tcttctgccgtgtcactagg-3' | 397bp | 60℃ |
| EXON3 | 5'-actaacgccctcttccttcc-3' | 5'-caagagggatactggcaggg-3' | 279bp | 60℃ |
| EXON4 | 5'-ccagtatccctcttggcaca-3' | 5'-cttttctgacccctgccct-3' | 483bp | 60℃ |
| EXON5 | 5'-agactctctccagggcttt-3' | 5'-cgagttcctgccatgcttc-3' | 299bp | 63℃ |
| EXON6 | 5'-caaggaagcatggcaggaac-3' | 5'-agtcacaccaaaccccagaa-3' | 366bp | 60℃ |
| EXON7 | 5'-atccgtgtgattcccgagg-3' | 5'-cgggaagggcatcatggaat-3' | 643bp | 60℃ |
| EXON8 | 5'-acctgcgaatcttctccctg-3' | 5'-gacacctgccttaccccttc-3' | 288bp | 60℃ |
| EXON9 | 5'-tctcctctctcctctgcagt-3' | 5'-tacttgtactggcgtgggtt-3' | 381bp | 60℃ |
| EXON10 | 5'-ctgctcagctcccttcctg-3' | 5'-tgttgtttcacttgtcctggg-3' | 371bp | 58℃ |
| EXON11 | 5'-ctgcttcttcggtcttctgtg-3' | 5'-ttggaactagaaggcatgcc-3' | 323bp | 60℃ |
| EXON12 | 5'-tgattgaaattggaggagggg-3' | 5'-gagtcaggacaggtgaggac-3' | 367bp | 60℃ |
| EXON13 | 5'-aatgtcaccctcacccacaa-3' | 5'-cacttgccacttcccctttc-3' | 324bp | 60℃ |
